# Phytochemical Compounds and Pharmacological Properties of *Larrea tridentata*

**DOI:** 10.3390/molecules27175393

**Published:** 2022-08-24

**Authors:** Ana Lizet Morales-Ubaldo, Nallely Rivero-Perez, Benjamín Valladares-Carranza, Alfredo Madariaga-Navarrete, Rosa Isabel Higuera-Piedrahita, Lucía Delgadillo-Ruiz, Rómulo Bañuelos-Valenzuela, Adrian Zaragoza-Bastida

**Affiliations:** 1 Área Académica de Medicina Veterinaria y Zootecnia, Instituto de Ciencias Agropecuarias, Universidad Autónoma del Estado de Hidalgo, Rancho Universitario Av. Universidad km 1, EX-Hda de Aquetzalpa, Tulancingo 43600, Hidalgo, Mexico; 2 Facultad de Medicina Veterinaria y Zootecnia, Universidad Autónoma del Estado de México, El Cerrillo Piedras Blancas, Toluca 50090, Estado de Mexico, Mexico; 3 Área Académica de Ciencias Agrícolas y Forestales, Universidad Autónoma del Estado de Hidalgo, Instituto de Ciencias Agropecuarias, Rancho Universitario Av. Universidad km 1, EX-Hda de Aquetzalpa, Tulancingo 43600, Hidalgo, Mexico; 4 Facultad de Estudios Superiores Cuautitlán, Universidad Nacional Autónoma de México, Carretera Cuautitlán-Teoloyucan km 2.5, San Sebastián Xhala, Cuautitlán 54714, Estado de Mexico, Mexico; 5 Unidad Académica de Medicina Veterinaria y Zootecnia, Universidad Autónoma de Zacatecas, Kilómetro 31.5 Carretera Panamerica, Fresnillo 98500, Zacatecas, Mexico

**Keywords:** bioactive compounds, pharmacological activities, *Larrea tridentata*

## Abstract

For centuries, traditional medicine from plants (phytotherapy) was the only treatment for infectious and non-infectious diseases. Although it is still practiced in several countries with excellent therapeutic results, it is frequently underestimated because, unlike Western medicine, it is not based on an empirical scientific foundation. However, interest in the search for plant-based therapeutic resources has been stimulated by disciplines such as phytochemistry and the side effects of conventional pharmacological therapies. For example, *Larrea tridentata* is a perennial shrub used in traditional medicine in northern Mexico and the southern United States to treat infertility, rheumatism, arthritis, colds, diarrhea, skin problems, pain, inflammation and excess body weight. Scientific research has revealed its beneficial effects—antioxidant, antitumor, neuroprotective, regenerative, antibacterial, antiviral, antifungal, anthelmintic, antiprotozoal and insecticidal—although reports indicate that some compounds in *Larrea tridentata* may be hepatotoxic and nephrotoxic. Therefore, the aim of this review was to highlight the updates regarding phytochemical compounds and the pharmacological properties of *Larrea tridentata.*

## 1. Introduction

*Larrea tridentata* is a perennial shrub of Mexico and the United States that is used to treat a variety of illnesses. This species belongs to the *Zygophyllaceae* family, comprising about 30 genera and 250 species and found mainly in warmer and drier regions (Table 1) [1,2,3].

*L. tridentata* is commonly known as chaparral and greasewood in the United States and guamis, fake caper, hediondilla and gobernadora in Mexico. It grows from 0.5 to 3.5 m and has little aroma. The stem has numerous branches with lanceolate green-yellowish leaves. Its flowers are yellow, and the fruit is ovoid with fine white hairs and contains a black seed [2,4].

This plant is well known in both Mexico and the USA for its effectiveness in treating a variety of illness: infertility, rheumatism, arthritis, diabetes, gall and kidney stones, colds, diarrhea, skin problems, overweight, pain and inflammation. It also has uses in industry and as forage. Some studies have centered on its bioactive compounds, mainly to evaluate its anti-inflammatory, antiviral, antifungal, antibacterial, antioxidant and neuroprotective properties [2,4,5,6,7,8]. The aim of this review was to highlight the updates regarding phytochemical compounds and the pharmacological properties of *Larrea tridentata.*

## 2. Methodology

To carry out the present review, a comprehensive search was performed in the following databases: PubMed, ScienceDirect and Google scholar for studies published from 2010 to 2022, since a previous literature review was published in 2009. The following headings and keywords were used: *Larrea tridentata*, gobernadora, creosote bush, bioactive compounds and biological activities. Duplicate papers were removed, the data were screened, irrelevant work was excluded and full-text documents were then screened. Inclusion criteria included several factors, involving original articles or reviews and work on natural or chemical compounds. Exclusion criteria were inadequate methods and lack of access to the full text.

## 3. Phytoconstituents

*L. tridentata* is a species rich in bioactive compounds—tannins, flavonoids, saponins, phytoestrogens and terpenes—and bioactive molecules: ellagic acid, gallic acid, catechins, methyl gallate, cinnamic acid resorcinol, kaempferol, quercetin, nordihydroguaiaretic acid (NDGA), thymol and carvacrol [9,10,11,12,13]. Table 2 shows the main active constituents isolated from *L. tridentata* [14,15,16,17,18,19,20,21,22,23,24].

### Isolation of New Compounds

In recent years, many studies have centered on elucidating new compounds. Jitsuno and Mimaki (2010) [25] performed a study that isolated 13 new compounds, identified as triterpene glycosides from the aerial parts of *L. tridentata*. For their part, Yokosuka et al. (2011) [26] isolated two new lignan glycosides called larrealignans. In a study carried out by Favela-Hernández et al. (2012) [27], a furanoid lignan from the leaves of *Larrea tridentata*, 4-epi-larreatricin, was isolated. Schmidt et al. (2012) [28], isolated nine lignans (dibenzylbutanes, epoxylignans and aryltetralins), six flavonoids and one ester.

Recently, two new cyclolignans were elucidated as 4,4′-dihydroxy-3-methoxy-6,7′-cyclolignan and 3,4-dihydroxy-3′,4′-dimethoxy-6,7′-cyclolignan [29]. Table 3 summarizes some of the new isolated compounds.

## 4. Pharmacological Activities

### 4.1. Antioxidant Activity

Antioxidant compounds are widely distributed in the plant kingdom, and in this regard, Martins et al. in 2010 evaluated the antioxidant capacity of *L. tridentata* through ferric reducing/antioxidant power (FRAP) and free radical-scavenging capacity techniques. They used 2,2-diphenyl-2-picrylhydrazyl (DPPH) radical-scavenging assays, and the data showed high antioxidant activity, which was attributed to elevated concentration of phenolic compounds and NDGA [30].

In a study performed by Rahman et al. (2011), the modulatory effects of *L. tridentata* and its associated compound NDGA were studied on acute inflammatory and oxidative stress responses in mouse skin induced by 12-*O*-tetradecanoylphorbol-13-acetate (TPA). They determined that pre-treatment with NDGA before the TPA application mitigated cutaneous lipid peroxidation and inhibited production of hydrogen peroxide. In addition, glutathione levels and antioxidant enzymes were restored, and the activity of myeloperoxidase and xanthine oxidase as well as skin edema formation were lowered [31].

In 2018, Aguirre-Joya et al. reported antioxidant capacity after evaluating ABTS^.+^ radical cation-scavenging activity assay, DPPH, lipid oxidation inhibition (LOI) and FRAP. The antioxidants identified were NDGA, quercetin and kaempferol [32].

Skouta et al. (2018) determined the antioxidant activity of three different extracts of *L. tridentata* (ethanol, ethanol–water and water), through DPPH, ABTS, superoxide, FRAP activity and nitric oxide (NO) assays, determining that ethanol–water (60:40) extract had the most efficient antioxidant properties, with values of 111.7 ± 3.8 µg/mL (DPPH), 8.49 ± 2.28 µg/mL (ABTS), 0.43 ± 0.17 µg/mL (superoxide) and 230.4 ± 130.4 µg/mL (NO). In addition, nine compounds were identified with antioxidant properties, among which were justicidin B and beta peltain [33].

Morán-Santibañez et al. (2019) reported that an ethanol–water extract from *L. tridentata* leaves mitigates cytotoxicity caused by oxidative stress in human cells. In addition, because of its cytoprotective activity against oxidative stress, the extract reduced the levels of different apoptosis hallmarks, thereby showing it to be a natural anti-apoptotic [34].

### 4.2. Antitumor Activity

The main compound of *Larrea tridentata* (NDGA) showed antitumor effects in bladder T24 cancer cells in vitro. The reactive oxygen species (ROS) levels were evaluated, and after 72 h of incubation, NDGA had reduced T24 cell viability in a dose-dependent manner. Apoptosis also increased at 48 h, and a dose of 20 μM of NDGA promoted mitochondrial stress by inducing oxygen consumption alterations just as in cancer cell death. This suggested that the antitumor effects of NDGA in T24 cells were related to its ability to induce mitochondrial alteration [35].

Probst et al. (2017) reported that lipoxygenase (LOX) inhibitors, such as NDGA, protect acute lymphoblastic leukemia (ALL) cells from RSL3-stimulated lipid peroxidation, reactive oxygen species generation (ROS) and cell death [36].

### 4.3. Neuroprotective Effects

NDGA has shown protective effects in the acute phase of stroke. In a 2014 transient ischemia rat model study carried out by Zhang et al., NDGA promoted neurogenesis and angiogenesis after 28 days of ischemia and reperfusion by suppressing semaphorin 3A expression [37].

Some studies have also centered on the use of natural products to treat neurodegenerative disorders, such as Alzheimer´s disease (AD) [38]. In this regard, Siddique and Ali (2017) evaluated the effect of NDGA on transgenic *Drosophila* expressing wild-type human Aβ-42 in the brain. Data showed that exposure to doses of 20, 40, 60 and 80 μM of NDGA reduced symptoms, increased life span, delayed the loss of climbing activity and showed a dose-dependent decrease in the activity of caspase 3 and 9 and acetylcholinesterase, which suggested an anti-apoptotic and neuroprotective role. Furthermore, NDGA improved memory loss in flies with AD, demonstrating that this compound reduced neurotoxic, motor and cognitive impairments [39].

### 4.4. Regenerative Applications

*Larrea tridentata* and its pure compounds have been widely used in different fields, including tissue engineering. Tovar-Carrillo et al. (2020) analyzed the compatibility of the in vitro and in vivo properties of cellulose hydrogels enriched with *L. tridentata* that had been implanted intramuscularly in female rats. At the end of the in vivo assay (90 days), no evidence of inflammation, toxicity or death was observed; furthermore, it was observed that the addition of *L. tridentata* improved cytocompatibility, demonstrating that enriched hydrogels can be used as regenerative scaffolds [40].

### 4.5. Hepatoprotective Effect

Del Vecchyo-Tenorio et al. (2016) stated that ethanolic extract of *L. tridentata* is useful in metabolic syndrome (MS) treatment since it was reported that the addition of the extract in a high fat and cholesterol diet (HFD) in hamsters with signs of MS reduced plasma triglycerides, total cholesterol, insulin and leptin and improved insulin sensitivity. On the other hand, in a standard diet enriched with the same extract, the effects were higher since reduced body and liver weight, glucose concentration, cholesterol, insulin and leptin in serum were increased in addition to insulin sensitivity. According to the authors, these effects were associated with lower lipid peroxidation and increased antioxidant capacity in the liver [41].

Chan et al. (2018), in a murine model with liver injury produced by the American Lifestyle-Induced Obesity Syndrome diet (ALIOS), demonstrated that the coadminstration of NDGA reduced body and epididymal fat weight and levels of alanine aminotransferase (ALT), aspartate aminotransferase (AST) and triglycerides in the liver. It also improved insulin sensitivity because this compound induced the activation of PPARα, a regulator of fatty acid oxidation, and the mRNA of Cpt1c and Cpt2, genes involved in fatty acid oxidation; furthermore, the NDGA reduced liver stress and the expression of (CASP3), an apoptosis signaling protein, and improved the hepatic expression of antioxidant enzymes and the proteins GPX4 and PRDX3 [42].

In a similar study, Han et al. (2019) evaluated the effect of NDGA (2.5 g/kg of diet) on mice fed a diet high in trans-fat, cholesterol and fructose (HTF) for 16 weeks. The NDGA reduced body and liver weight and the liver-to-body weight ratio in HTF-fed mice also decreased non-esterified fatty acids and serum insulin. The results suggested that NDGA could mitigate liver damage and the accumulation of triglycerides. A glucose tolerance test revealed that mice treated with NDGA showed lower levels of glucose, steatosis and fibrosis. Furthermore, this compound increased fatty acid oxidation and reduced both ER and oxidative stress [43].

### 4.6. Renal Effects

Some studies have evaluated the renal effects of the major compound of *L. tridentata*. Zuntilde et al. (2012) reported that NDGA prevented renal dysfunction, histological damage and oxidative stress, as well as decreasing the activity of the renal antioxidant enzymes glutathione peroxidase, glutathione reductase, glutathione-S-transferase and catalase. It also affected mitochondrial activity, which is why NDGA was considered to be nephroprotective [44].

Zúñiga-Toalá et al. (2013) [45] reported that pretreatment with NDGA had a protective effect on ischemia–reperfusion renal (I/R) damage. It attenuated tubular epithelium damage since this compound induced, in vivo and in vitro, nuclear factor erythroid 2-related factor 2 (Nrf2) nuclear translocation in rats that had a uni-nephrectomy and I-R damage and apoptosis. The authors of this study suggest that the indirect antioxidant effect of NDGA may have been involved in the cytoprotective effect of the I-R injury, and previous studies support this result. Rojo et al. (2012) reported that NDGA increased the level of the Nrf2 protein and expression of heme oxygenase-1 (HO-1) in kidney cells through the activation of multiple signaling cascades [46].

### 4.7. Anti-Inflammatory Activity

In an in vivo study carried out by Rahman et al. (2011), mice treated with NDGA (15 and 25 μmol) before a double application of 12-*O*-tetradecanoylphorbol-13-acetate (TPA), showed significantly reduced activity of myeloperoxidase, one of the main enzymes related with polymorphonuclear (PMN) activation. It was also observed that animals treated with this compound showed a lower edema response compared with those treated only with TPA. Histological findings showed that the TPA application caused an increase in the epidermal layer, the infiltration of polymorphonuclears (PMNs) and intercellular edema in the skin. It also caused inflammatory responses in the tissue in contrast to those animals that had been pretreated and treated with NDGA, which mitigated inflammation and any histological change [31].

Xue et al. (2013) evaluated the in vivo anti-inflammatory effects of NDGA in spinal cord injury (SCI). Myeloperoxidase (MPO) levels were measured after 3 days of the SCI process, and the results showed that NDGA reduced neutrophil infiltration after injury and infiltration of macrophages–microglia. In this study, NDGA decreased inflammatory factors (IL-1β and TNF-α) associated with spinal cord damage [47].

### 4.8. Growth Performance

García-López et al. (2018) evaluated the effects of the dietary addition of whole plant, leaves and powdered aqueous extract of *L. tridentata* on the growth, organ weight and serum hepatic enzymes of Cobb broiler chickens. The treatments were added to a basal diet and randomly assigned to 200 Cobb broilers one day old. The authors concluded that those fed the *L. tridentata* aqueous extract had a better performance response; furthermore, the decrease in enzyme hepatic levels means that the extract could be considered a natural growth promoter [48].

In a similar study, dried aerial parts of *L. tridentata* were added to a sheep diet at a rate of 0, 5 and 10% over 60 days. An analysis indicated that the aerial parts contained 85% dry matter, 12% crude protein, 58% neutral detergent fiber and 10% ash, which was similar to hay or conventional silage. The pH values were similar to that of the control group. Despite the data in feed efficiency, there was no significant statistical difference between diets with and without the *L. tridentata* biomass. The study concluded that inclusion of *L. tridentata* in the diet may be suitable for finishing [49].

### 4.9. Hypoglycemic Effects

Roškar et al. (2016), reported that NDGA demonstrated antidiabetic activity in vivo since this compound inhibited α-amylase, α-glucosidase and dipeptidyl peptidase 4, enzymes associated with postprandial glucose management [50]. The pharmacological activities of *L. tridentata* and its related bioactive compounds are summarized in Table 4.

### 4.10. Antibacterial Activity

Seven compounds from the chloroformic *L. tridentata* extract were isolated and identified: dihydroguaiaretic acid; 4-epi-larreatricin and 3′-demethoxy-6-*O*-demethylisoguaiacin (lignans) and 5,4′-dihydroxy-3,7,8,3-tetramethoxyflavone, 5,4′-dihydroxy-3,7,8-trimethoxyflavone, 5,4′-dihydroxy-7-methoxyflavone and 5,8,4′-trihydroxy-3,7-dimethoxyflavone (flavonoids). All of these were evaluated through the determination of minimal inhibitory concentration (MIC) against Gram-negative (*Stenotrophomona maltophilia*, *Escherichia coli*, *Acinobacter baumannii*, *Haemophilus influenzae*, *Pseudomonas aeruginosa*, *Klebsiella pneumoniae* and *Enterobacter cloacae*) and Gram-positive (*Staphylococcus aureus*, *S. aureus* (MR), *Streptococcus pneumoniae*, *Listeria monocytogenes* and *Enterococcus faecalis*) bacteria. The results showed that six of the compounds had antibacterial activity in a range of concentrations from 12.5 to >50 µg/mL, with the most active compound being 3′-demethoxy-6-*O*-demethylisoguaiacin. It showed antibacterial activity against all evaluated bacteria; therefore, it was evaluated against clinical isolates of *E. faecalis*, *S. aureus* and *S. aureus* (MR) and attained MIC values from 12.5 to 50 µg/mL. The mechanism of action of this compound affected the proteins of the ATP-binding cassette (ABC) transport system, thereby causing bacteria death [27,51].

In a study performed by Mendez et al. (2012) [52], different *L. tridentata* leaf extracts (water, ethanol, cocoa butter and lanolin) were evaluated against *E. aerogenes*, *E. coli*, *S. typhi* and *S. aureus*. The results showed that ethanolic extract had the highest growth inhibitory effects on *E. coli* and *S. aureus*. Snowden et al. (2014) evaluated *L. tridentata* leaf and flower extracts against *S. aureus* and obtained an MIC of 60 µg/mL, demonstrating that the extracts had bacteriostatic and bactericidal activity [53].

For their part, Martins et al. (2013) [54] evaluated the antibacterial activity of *L. tridentata* crude methanolic (CME) extract, hexane (H), dichloromethane (DCM), ethyl acetate (EA) and ethanol (Et) fractions and the compound NDGA. Antibacterial activity was determined through agar diffusion, and the results showed that CME, DCM, EA and NDGA were active against Gram-positive bacteria (*S. aureus*, *S. aureus* methicillin-resistant (MRSA), *Staphylococcus saprophyticus*, *Staphylococcus epidermidis* and *Enterococcus faecalis*). An MIC from 31.3 to 125 µg/mL was obtained for the EA fraction, the most active, and 31.3 µg/mL for MRSA, the most sensitive bacterium, which was at a concentration lower than the reference antibiotic, tetracycline (64 µg/mL). In addition, the authors identified three bioactive compounds: quercetin, kaempferol and NDGA, all of which had reported antibacterial activity [54].

The combination of NDGA and conventional antibiotics (gentamicin, neomycin and tobramycin) showed synergistic activity (97–100%) against 200 clinical isolations of methicillin-sensitive *S. aureus* (MSSA) and methicillin-resistant *S. aureus* (MRSA). In addition, when the MIC values of these combinations were determined, all antibiotics were reduced 2 to 128-fold against MSSA and 2 to 256-fold against MRSA. Moreover in the time-kill assay, NDGA improved the effect of three antibiotics in in vitro and in vivo murine models. According to the authors, the enhancement of antibiotic efficacy was due to the ability of NDGA to permeabilize bacterial membranes [55].

The antibacterial effects of thymol and carvacrol in the *L. tridentata* ethanolic extract were demonstrated in a study carried out by Delgadillo-Ruiz et al. (2017) [12]. Meso-dihydroguaiaretic acid derivatives (esters, ethers and amino-ethers) were tested against Gram-positive and Gram-negative drug-resistant bacterial strains, showing that Gram-positive bacteria (MR *S. aureus*, VR *E. faecium*, LR *S. epidermis* and LR *S. haemolyticus*) were more sensitive, and even two amino-ethers were more active than levofloxacin [56].

A direct comparison of the antibacterial activity against nonantibiotic-resistant *S. aureus* and two different strains of antibiotic-resistant *S. aureus* was performed by Gerstel et al. (2018) [57]. They determined an MIC range of 0.35–15 µg/mL for *L. tridentata* extract. In 2019, Itza-Ortiz stated that *L. tridentata* extract at 30% generated bacterial growth inhibition halos (BGIHs) against a wide range of Gram-negative and Gram-positive bacteria (0.67–1.73 mm). The extract was the most active against *S. aureus* and *S. enterica* when the BGIHs were 1.73 and 1.57 mm, respectively [58].

From the chloroform extract of *L. tridentata*, the compounds 4,4′-dihydroxy-3-methoxy-6,7′-cyclolignan, 3,4-dihydroxy-3′,4′-dimethoxy-6,7′-cyclolignan, meso-dihydroguaiaretic acid, 3′-demethoxyisoguaiacin, 3′-demethoxy-6-*O*-demethylisoguaiacin, nordihydroguaiaretic acid, 5,4′-dihydroxy-3,7,8-trimethoxyflavone and 5,8,4′-trihydroxy-3,7-dimethoxyflavone were isolated. These were active against nine multidrug-resistant clinical isolates at concentrations from 6.25 to >50 µg/mL [29]. In a further study, seven amino-ether derivatives from lignan 4,4′-dihydroxy-3-methoxy-6,7′-cyclolignan exhibited antibacterial activity against Gram-positive bacteria [59].

Turner et al. (2021) determined that the ethanolic extract of *L. tridentata* showed bactericidal activity against *S. aureus* (20 μg/mL), *S. pyogenes* (30 μg/mL), *B. cereus* (120 μg/mL), *E. coli and P. aeruginosa* (>1000 μg/mL); moreover, the authors determined that *L. tridentata* extract enhanced the activity of some β-lactam antibiotics, suggesting the presence of a β-lactam-type antibiotic in the extract [60].

Recently, Morales-Ubaldo et al. (2022) evaluated the antibacterial activity of a hydroalcoholic extract, fractions (aqueous and ethyl acetate) and subfractions from organic fractions, all of which were derived from *L. tridentata* aerial parts. When measured against the reference and multidrug-resistant bacterial strains associated with bovine mastitis, the data showed that the antibacterial activity of *L. tridentata* was associated with the pure compound nor 3’-demethoxyisoguaiacin, which exhibited the highest bactericidal effects [61].

### 4.11. Antimycobacterial Activity

In 2018, according to the World Health Organization (WHO), 1.5 million people died from tuberculosis (TB), one of the top 10 causes of death; moreover, multidrug-resistant TB (MDR-TB) represents a public health threat [62].

In this respect, studies have centered on the search for agents capable of acting against this bacteria. Favela-Hernández et al. (2012) tested seven compounds against both sensitive and MDR *Mycobacterium tuberculosis* strains and obtained an MIC of 12.5 to >50 µg/mL. The compounds responsible for this activity were dihydroguaiaretic acid 4-epi-larreatricin, 3′-demethoxy-6-*O*-demethylisoguaiacin, 5,4′-dihydroxy-3,7,8,3-tetramethoxyflavone and 5,4′-dihydroxy-3,7,8-trimethoxyflavone, [27].

In 2014, Clemente-Soto et al. found that a concentration of 50 µg/mL of meso-dihydroguaiaretic acid (MDGA) inhibited bacterial growth after 48 h [63]; in a similar study, Reyes-Melo et al. (2017) [56] found that MDGA derivatives affected sensitive and multidrug-resistant *M. tuberculosis* strains; MIC values ranged from 3.125 to 50 µg/mL. Furthermore, the authors determined that this compound had no cytotoxic effects. Guzmán-Beltrán et al. (2016) determined that a concentration of 250 µg/mL of NDGA exerted bactericidal activity [64].

The study carried out by Nuñez-Mojica et al. (2021) determined that eight compounds isolated from *L. tridentata* leaves exhibited activity against a susceptible and drug-resistant *M. tuberculosis* strain, and in a further study, amino-ether derivatives from lignan 4,4′-dihydroxy-3-methoxy-6,7′-cyclolignan exhibited antitubercular activity. The most active the compound against the multidrug-resistant *M. tuberculosis* strain was identified as 4C (6.25 µg/mL) [29,59].

### 4.12. Antiviral Activity

The in vitro antiviral activity of the methylated derivative of NDGA, terameprocol (TMP), was tested to determine if it could inhibit poxvirus (CPXV) growth. The authors performed CPXV plaque-reduction assays containing varied concentrations of TMP: 3.125, 6.25 and 12.5 µM. The results showed a dose-dependent decrease in CPXV plaque size and a reduction in the total number of plaques that could be detected. It was reported that the compound inhibited poxvirus growth in vitro by preventing the efficient spread of virus particles from cell to cell [65].

### 4.13. Antiprotozoal Activity

In the study carried out by Schmidt et al. (2012), dichloromethane extract from aerial parts of *L. tridentata* was used for antiprotozoal screening against *Trypanosoma brucei rhodesiense*, *Trypanosoma cruzi*, *Leishmania donovani* and *Plasmodium falciparum*, which had IC_50_ values of 2.8, 14.6, 5.2 and 2.9 µg/mL, respectively. Nine lignans, six flavonoids and one ester of ferulic acid were isolated and evaluated. Lignan meso-nordihydroguaiaretic acid showed the majority of activity obtaining IC_50_ of 4.5, 33.1, 12.0 and 7.7 µM against the above-mentioned parasites, respectively [28].

In their study, Camacho-Corona et al. (2015), evaluated the organic extracts of six plants, including *L. tridentata* against *Entamoeba histolytica*, *Giardia lamblia* and *Trichomonas vaginalis* and obtained IC_50_ values of 100, 116 and 118 µg/mL, respectively [66].

It was also reported that *Entamoeba histolytica*, *Giardia lamblia*, and *Naegleria fowleri* are susceptible to six lignan compounds from *L. tridentata.* Compound 1 (NDGA) showed the highest activity against *G*. *lamblia* and *N*. *fowleri* (EC_50_ 36 and 37 μM, respectively), and moderate activity against *E. histolytica (*EC_50_ =103 μM); compound 2 (3′-*O*-methyl-NDGA) showed similar results to compound 1 against *N*. *fowleri* (EC_50_ 38 μM). In both cases these results were better than the standard drug (miltefosine EC_50_ = 54.5 μM). The other compounds showed activity from 49 to 235 μM. In addition, the authors suggested that the activity of compounds 1 and 2 against *N*. *fowleri* may be due to the modulation of cysteine protease activity in the trophozoites [67].

### 4.14. Anthelmintic Activity

Regarding parasitic infections in sheep, the anti-*Haemonchus contortus* properties of *L. tridentata* were reported by García et al. (2018). Sheathed and unsheathed worm larvae of *H. contortus* were incubated with hydro-methanolic extract at concentrations of 12.5, 25, 50, 100 and 200 mg/mL during 24, 48 and 72 h. At the highest concentration, the extract showed weak activity against sheathed larvae (30% mortality), but against unsheathed larvae activity increased to 70%; moreover, the authors found that the compounds identified in their study damaged the larval cuticle and that the worms coiled up and were lethargic [68].

### 4.15. Antifungal and Antibacterial Activity in Agricultural Crops

*L. tridentata*-lanolin, cocoa butter and water extracts were evaluated against *Rhizoctonia solani*, an agent of diseases associated with roots and tubers of different crops. The data showed that *L. tridentata*-lanolin extract at 500 ppm of total tannins inhibited 80% of mycelia, but when the tannins were increased to 2000 ppm, 100% inhibition was obtained. Cocoa butter extract required a concentration of 3000 ppm of total tannins to obtain the same inhibition percentage (100%), and water extract required 8000 ppm [69].

In the 2010 study carried out by Osorio et al., polyphenolic extract from *L. tridentata* leaves was evaluated against *Pythium* spp., *Colletotrichum truncatum*, *Colletotrichum coccodes*, *Alternaria. alternata*, *Fusarium verticillioides*, *Fusarium solani*, *Fusarium sambucinum* and *Rhizoctonia solani*, which are all associated with leaf and root diseases. Strong fungicidal activity was observed since the extract inhibited 100% of the fungal strains, except for *F. verticillioides*, for which inhibition was 75%; moreover, the extract was evaluated against 10 different single-spore isolates of *Fusarium oxysporum*. *L. tridentata* inhibited eight of them 100%, and one 75% [70].

Chávez-Solíz et al. (2014) reported that *L. tridentata* leaf extracts at concentrations of 1000 and 5000 mg/L of water, significantly reduced the severity of *Podosphaera xanthii*, one of the causative agents of powdery melon mildew [71]. Galván et al. (2014) reported that the best antifungal activity of *L. tridentata* aqueous extract (10 and 20%) was against *Phytophthora capsici* and *Aspergillus flavus*. After 48, 72 and 96 h, the two extract concentrations caused 100%inhibition in both fungal species [72].

*L. tridentata* leaf extract alone or in combination with potassium sorbate had positive effects against *A. flavus* in pH conditions 3, 4 and 5. Inhibited growth of 71.91, 69.33 and 70.06% (pH 3, 4 and 5, respectively) was achieved at a concentration of 1000 ppm. It increased to 81.48, 82.82 and 81.43% when the potassium sorbate was added in the same respective pH conditions. The authors determined that, together, both compounds demonstrated synergistic activity [73].

The in vitro antifungal activity of *L. tridentata* water, ethanol, lanolin and cocoa butter extracts against *Phytophthora cinnamomi* Rands was evaluated in a 2015 study by Castillo-Reyes et al. The data showed that ethanol extract caused 100% mycelium inhibition, and lanolin extract caused 80%. When MIC_50_ was determined, a concentration of 6.96 ppm was needed to inhibit 50% of mycelia growth; for lanolin extract, it was 183.6 ppm [74].

Peñuelas-Rubio et al. (2015) [75] reported that *L. tridentata* ethanolic and dichloromethane extracts inhibited fungal growth 75–100% against *Alternaria tenuissima*, *Aspergillus niger*, *Penicillium polonicum* and *Rhizopus oryzae*. In a similar study, ethanolic and dichloromethane extracts inhibited *Fusarium oxysporum radicis-lycopersici* 100%, while methanolic extract achieved 94% [76].

Aguirre-Joya et al. (2018) reported high fungistatic activity by using a bioactive film containing *L. tridentata* polyphenols, which achieved MIC_50_ values of 566, 558, 612 and 579 ppm for *Alternaria alternata*, *Fusarium oxysporum*, *Botrytis cinereal* and *Colletotrichum gloeosporioides*, respectively [32].

Recently, Morales-Ubaldo et al. (2021), reported the antibacterial activity of a hydroalcoholic extract and ethyl acetate fraction of *L. tridentata*, against multidrug-resistant phytopathogenic bacteria (*Clavibacter michiganensis* subsp. *michiganensis*, *Pseudomonas syringae* and *Xanthomonas campestris*). The authors found that the extract showed inhibitory activity at concentrations of 0.39–6.25 mg/mL and bactericidal effects at 0.78–12.5 mg/mL. The concentrations of ethyl acetate fraction were 0.39–3.12 and 0.78–6.25 mg/mL for MIC and MBC, respectively. According to the authors, their study is the first to report the antibacterial activity of *L. tridentata* against multidrug-resistant phytopathogenic bacteria [77].

For their part, Méndez-Andrade et al. (2021) used an aqueous extract from *L. tridentata* leaves as a source for reducing and stabilizing agents to obtain silver nanoparticles, which exert bactericidal activity against *Clavibacter michiganensis*. The authors reported that, at a concentration of 50 mg/L, disease incidence did not exceed 20%, and disease severity was reduced by 36% [78].

### 4.16. Insecticidal Activity

Pecan black aphids (*Melanocallis caryaefoliae* D.) were exposed to *L. tridentata* (stem and leaf) ethyl acetate, methanol, and water extracts at concentrations of 0.5, 1 and 2%. The authors determined that leaf extracts at 1% concentration showed 80 and 92% mortality in the aqueous and methanol extracts, respectively. Regarding stem extracts, the best effect was obtained when ethyl acetate and aqueous extracts (0.5%) were used. For all extracts, the greatest effect occurred 72 h after the treatment was applied. In addition, the repellent effect was evaluated and showed the best effects after 24 h: 65% repellence from 0.5% methanolic leaf extract; however, 1% ethyl acetate stem extract showed the best activity, repelling 50% of the pecan black aphids [79].

A similar study reported that 20% *L. tridentata* leaf extract reduced the incidence of horn flies (*Haematobia irritans*) on cows [80]. The larvicidal effect of *L. tridentata* extract (1 g/L) on mosquitoes was approximately 50% mortality (L3) [81]. Some of the pharmacological effects of *L. tridentata* related to infectious diseases are shown in Table 5.

## 5. Side Effects

There are reports stating that the products of *L. tridentata* may be associated with jaundice, cholestatic hepatitis and liver damage, which then progresses to cirrhosis and even fulminant liver failure. In addition, the main compound, NDGA, causes hepatotoxicity and nephrotoxicity in humans and death in mice (LD_50_ = 75 mg/kg). Contact dermatitis has also been attributed to *L. tridentata* [8,82,83,84,85,86].

## 6. Discussion and Future Prospects

*L. tridentata* has traditionally been used to treat a variety of diseases. In recent years several investigations have demonstrated the pharmacological properties of this botanical species, especially its antimicrobial activity. Before determining its effects in vitro on bacteria (human, animal, or plant), parasites, fungi and viruses, it is necessary to perform in vivo or in situ tests to support the efficacy of *L. tridentata* as an effective alternative method of treatment. In the same sense, further studies are needed to establish new strategies to improve its pharmacological properties and phytochemical content, such as the study by Nuñez-Mojica et al. (2022), in which the derivation of a known compound from *L. tridentata* led to 11 new antibacterial compounds [59]. In the same sense, is necessary to establish a dose-response relationship for extracts, fractions or pure compounds associated with their toxicological profile and their mechanism of action.

## 7. Conclusions

This review examined the pharmacological effects of *L. tridentata*, commonly known as gobernadora in Mexico and creosote bush in the USA. It was found that the aerial parts of *L. tridentata* are of great importance in both traditional medicine and pharmaceuticals for treatment of infectious and non-infectious diseases because of their antioxidant, neuroprotective, antitumoral, anti-inflammatory, regenerative, antifungal, insecticidal, anthelmintic, antiprotozoal and antibacterial activities. These are associated with such bioactive compounds as ellagic acid, gallic acid, catechins, methyl gallate, cinnamic acid resorcinol, kaempferol, quercetin, nordihydroguaiaretic acid (NDGA), thymol and carvacrol. However, nephrotoxic and hepatotoxic effects, mainly associated with NDGA, have been reported.

## Figures and Tables

**Table 1:** Taxonomic classification of *Larrea tridentata*.

Taxonomy	
Kingdom	*Plantae*
Division	Tracheophyta
Class	Magnoliopsida
Order	Zygophyllales
Family	Zygophyllaceae
Genus	*Larrea*
Species	*Tridentata*

**Table 2:** Chemical structure of bioactive molecules isolated from *L. tridentata* from the International Union of Pure and Applied Chemistry (IUPAC).

Compound	Class of Compound	IUPAC Name	Chemical Structure
Ellagic acid	organic heterotetracyclic compound, polyphenol	6,7,13,14-tetrahydroxy-2,9-dioxatetracyclo [6.6.2.0^4,16^.0^11,15^]hexadeca-1(15),4,6,8(16),11,13-hexaene-3,10-dione	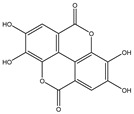
Gallic acid	trihydroxybenzoic acid	3,4,5-trihydroxybenzoic acid	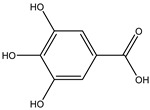
Catechins	Hydroxyflavanoids	2-(3,4-dihydroxyphenyl)-3,4-dihydro-2*H*-chromene-3,5,7-triol	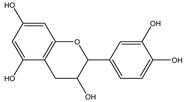
Methyl gallate	Gallate ester	methyl 3,4,5-trihydroxybenzoate	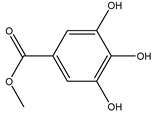
Cinnamic acid	Monocarboxylic acid, a styrene	(*E*)-3-phenylprop-2-enoic acid	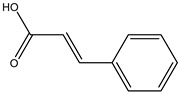
Resorcinol	Benzenediol	benzene-1,3-diol	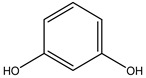
Kaempferol	Flavonol (tetrahydroxyflavone)	3,5,7-trihydroxy-2-(4-hydroxyphenyl)chromen-4-one	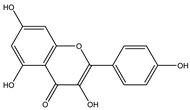
Quercetin	Flavonoid	2-(3,4-dihydroxyphenyl)-3,5,7-trihydroxychromen-4-one	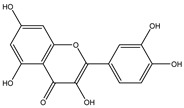
Nordihydroguaiaretic acid (NDGA)	Lignan	4-[4-(3,4-dihydroxyphenyl)-2,3-dimethylbutyl]benzene-1,2-diol	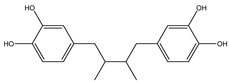
Thymol	Monoterpene	5-methyl-2-propan-2-ylphenol	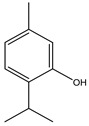
Carvacrol	Monoterpene	2-methyl-5-propan-2-ylphenol	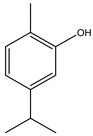

**Table 3:** Isolated compounds from *Larrea tridentata*.

Organ-Extract	Compound	Class of Compound
Aerial parts, methanolic extract	3-[(*O*-(4-*O*-sulfo-b-d-glucopyranosyl)-(1→3)-a-L-arabinopyranosyl) oxy]olean-12-en-28-oic acid b-d-glucopyranosyl ester sodium salt 3-[(*O*-(4-*O*-sulfob-d-glucopyranosyl)-(1→3)-*O*-[a-l-rhamnopyranosyl-(1→2)]-a-Larabinopyranosyl)oxy]-30-noroleana-12,20(29)-dien-28-oic acid b-d-glucopyranosyl ester sodium salt	Triterpene glycosides
Aerial parts, methanolic extract	Larrealignans A and B	Lignans
Leaves, chloroformic extract	dihydroguaiaretic acid, 4-epilarreatricin, 3′-demethoxy-6-Odemethylisoguaiacin,	Lignans
Leaves, chloroformic extract	5,4′-dihydroxy-3,7,8,3′-tetramethoxyflavone 5,4′-dihydroxy-3,7,8-trimethoxyflavone 5,4′-dihydroxy-7-methoxyflavone 5,8,4′-trihydroxy-3,7-dimethoxyflavone	Flavonoids
Aerial parts, dichloromethane extract	3,4-dehydrolarreatricin meso-dihydroguaiaretic acid 3-*O*-methyldihydroguaiaretic acid 3-*O*-demethylisoguaiacin	Lignans
Aerial parts, dichloromethane extract	3′-oxohexyl ferulate	Ferulic acid ester
Aerial parts, dichloromethane extract	Naringenin 3′-*O*-methyltaxifolin apigenin-7-methylether Kaempferol-3,7-dimethylether herbacetin-3,7-dimethylether	Flavonoids
Leaves, hexane extract	4,4′-dihydroxy-3-methoxy-6,7′-cyclolignan 3,4-dihydroxy-3′,4′-dimethoxy-6,7′-cyclolignan	Cyclolignans

**Table 4:** Pharmacological activities of *L. tridentata* its related compounds and mechanism of action.

Activities	Bioactive Compounds	Mechanism of Action	Reference
Antioxidant	NDGA, Quercetin, Kaempferol, Justicidin B and Beta peltain	Mitigation of cutaneous lipid peroxidation and cytotoxicity, inhibition of production of hydrogen peroxide and edema formation, reduction of apoptosis hallmarks	[30,31,32,33,34]
Antitumor	NDGA	Induction of mitochondrial alterations, ferroptosis.	[35,36]
Neuroprotective	NDGA	Promotion of neurogenesis and angiogenesis, anti-apoptotic, reduction of the neurotoxic, motor and cognitive impairments of Alzheimer´s disease	[37,38,39]
Regenerative	Not indicated	Inhibition of inflammation or toxicity	[40]
Hepatoprotective	NDGA	Lower lipid peroxidation, increase in antioxidant capacity in the liver	[41,42,43]
Renal effects	NDGA	Decreasing the activity of renal antioxidant enzymes, affection of mitochondrial activities.	[44,45,46]
Anti-inflammatory	NDGA	Reduction in myeloperoxidase activity, reduced edema response, decrease of inflammatory factors	[31,47]
Hypoglycemic	NDGA	Inhibition of α-amylase, α-glucosidase and dipeptidyl peptidase 4	[50]

**Table 5:** Pharmacological activities of *Larrea tridentata* related to infectious diseases.

Activities	Bioactive Compound	Mechanism of Action	Reference
Antibacterial activity	Several bioactive Compounds	Affecting proteins of ABC transport system causing bacteria death, bacterial retardation, bacteriostatic, permeabilizing membrane	[12,27,51,52,53,54,55,56,58,59,60,61]
Antimycobacterial activity	Lignans, flavonoids, meso-dihydroguaiaretic acid, NDGA	Growth inhibition, bactericidal	[27,55,58,59,64]
Antiviral activity	Terameprocol (TMP)	Inhibition of poxvirus growth	[65]
Antiprotozoal activity	Several compounds	Modulation of cysteine protease activity present in the trophozoites	[28,66,67]
Anthelmintic activity	Hydro methanolic Extracts	Damaging larvae cuticle, coiling up of worms and lethargic movements	[68]
Antifungal activity	Tannins, polyphenolic extracts	Fungi-static and fungicidal effects	[31,69,70,71,72,73,74,75,76]
Insecticidal activity	Different extracts	Repellent effect, causing death of mosquito larvae	[79,81]

## Data Availability

Data are contained within the article.
